# Pharmacokinetic and pharmacodynamic sub-study of trans-arterial vs. intravenous gemcitabine in the TIGeR-PaC phase 3 clinical trial

**DOI:** 10.1007/s00280-026-04939-0

**Published:** 2026-08-17

**Authors:** Paula M. Novelli, Emmanuel E. Zervos, Amer H. Zureikat, Michael J. Pishvaian, Kenneth Meredith, Hassan Hatoum, Reza Nazemzadeh, Sandeep Laroia, Pashtoon Kasi, Justus Bingham, Ramtin Agah

**Affiliations:** 1 University of Pittsburgh Medical Center, Pittsburgh, PA USA; 2 Brody School of Medicine at East Carolina University, Greenville, NC USA; 3 Johns Hopkins University School of Medicine, Washington, DC USA; 4 Sarasota Memorial Health Care System, Sarasota, FL USA; 5 University of Oklahoma College of Medicine, Oklahoma City, OK USA; 6 Levine Cancer Institute, Charlotte, NC USA; 7 University of Iowa Carver College of Medicine, Iowa City, IA USA; 8 City of Hope, Irvine, CA USA; 9 RenovoRx, Mountain View, CA USA; 10 Division of Surgical Oncology, Brody School of Medicine at East Carolina University, 600 Moye Boulevard, Greenville, 27834 NC USA

**Keywords:** Intra-arterial chemotherapy, Gemcitabine, Locally advanced pancreatic cancer (LAPC), Trans-arterial micro perfusion (TAMP)

## Abstract

**Purpose:**

Localized intra-arterial delivery of gemcitabine (IAG) may provide advantages in terms of increased tissue concentration and decreased systemic dosing. We hypothesized IAG may result in decreased systemic gemcitabine concentration and associated side effects due to intracellular delivery prior to conversion to gemcitabine’s inactive metabolite, difluorodeoxyuridine (dFdU). Here we report the results of a pharmacokinetics and pharmacodynamics sub-study within the TIGeR-PaC phase 3 clinical trial (NCT03257033).

**Methods:**

Analyses were performed for 16 participants across 6 TIGeR-PaC study sites; 11 participants received localized IAG and 5 participants received systemic intravenous gemcitabine (IVG). Gemcitabine and dFdU assays were performed on blood samples collected immediately before, during, and after infusion. CA 19 − 9 levels were measured prior to and 2 weeks after IAG treatment. Maximum plasma drug concentration (C_max_) and the area under the drug plasma concentration curve (AUC_0−t_) were compared between treatment groups. Pearson’s correlation for AUC_0−t_ and the percent change in CA 19 − 9 levels was calculated for the IAG group.

**Results:**

IAG resulted in lower gemcitabine C_max_ and AUC_0−t_ and higher dFdU C_max_ and AUC_0−t_ compared to IVG, consistent with a more rapid conversion of gemcitabine to dFdU with IAG versus IVG. With IAG, there was a significant correlation between increased dFdU levels and a pre- to post-treatment reduction in CA 19 − 9 levels (Pearson’s *r* = -0.75; *P* = 0.034).

**Conclusion:**

In addition to providing increased local potency, localized IAG, in which gemcitabine is rapidly converted to its inactive metabolite dFdU, may also be beneficial in decreasing gemcitabine-related systemic side effects.

## Introduction

Pancreatic cancer remains one of the most difficult cancers to treat, as it is generally identified after either local invasion of surrounding tissue or distant metastases have occurred. Locally advanced pancreatic cancer (LAPC) is characterized by tumor involvement of adjacent blood vessels or nearby organs. Localized disease accounts for approximately 50% of new pancreatic cancer diagnoses; for the majority of these, resection is precluded due to tumor involvement of the celiac axis, the superior mesenteric artery (SMA), or the hepatic artery [1, 2]. Thus, current LAPC treatment options are typically limited to systemic chemotherapy with or without subsequent chemoradiation or stereotactic body radiation therapy (SBRT). As the median overall survival rate for patients with LAPC is only 11–16 months even with systemic chemotherapy [3, 4], more effective treatment options are critically needed.

**Fig. 1:**
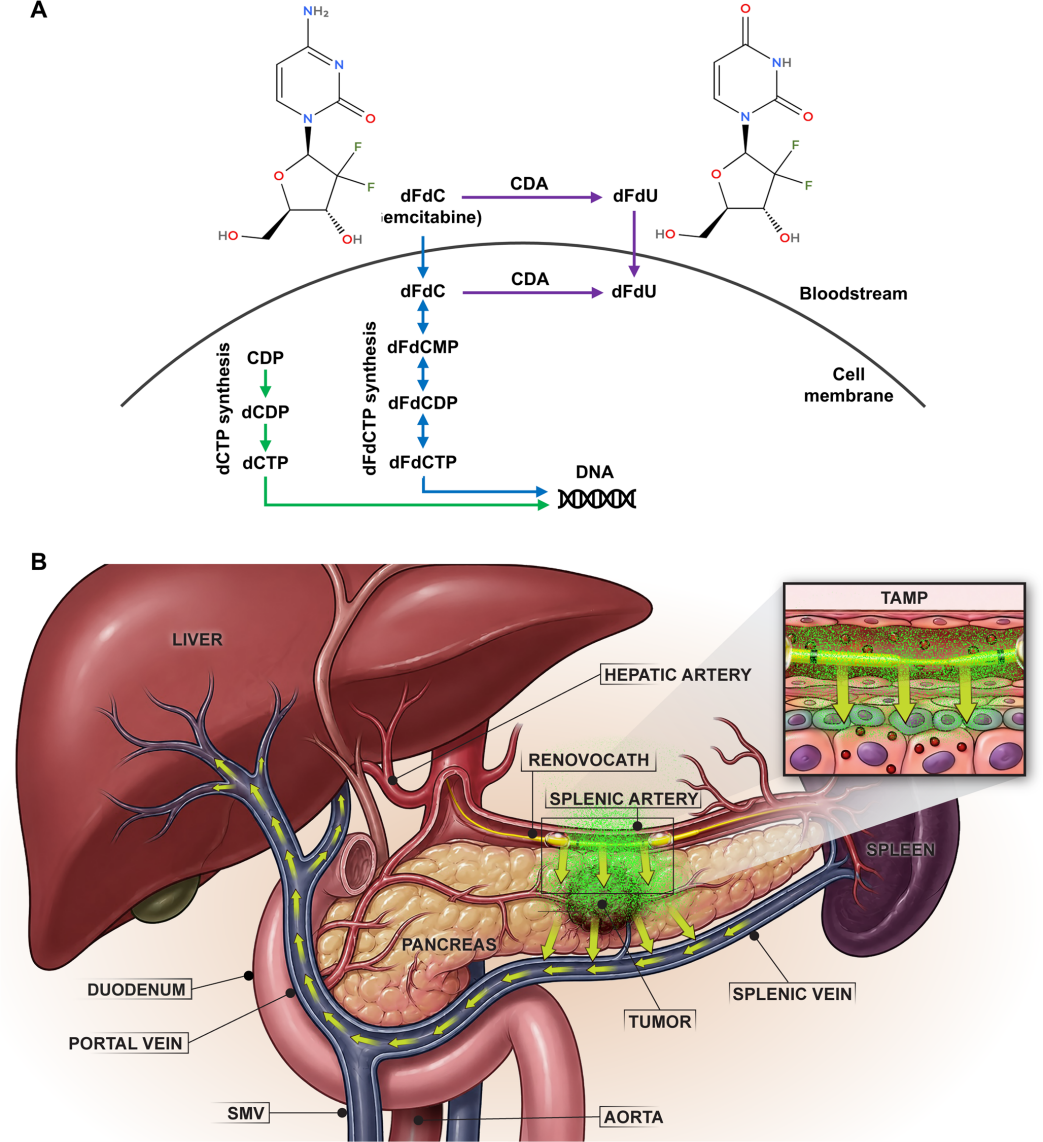
Gemcitabine metabolism and trans-arterial micro perfusion (TAMP)-mediated delivery. (**A**) Gemcitabine is converted to its inactive metabolite 2’,2’-difluorodeoxyuridine (dFdU) both in the blood and in tissue via cytidine deaminase (CDA). Alternatively, intracellular gemcitabine can undergo phosphorylation to its active metabolites gemcitabine diphosphate (dFdCDP) and gemcitabine triphosphate (dFdCTP). dFdCDP inhibits ribonucleotide reductase, thereby acting to starve the cell of necessary deoxyribonucleotide pools. dFdCTP competes with cytidine triphosphate (dCTP) for incorporation into DNA; once incorporated, dFdCTP acts as a chain terminator, precluding further DNA synthesis. (**B**) During TAMP-mediated drug delivery, the RenovoCath double-balloon cathether is introduced to the splenic artery, occluding blood flow in a segment adjacent to the pancreatic tumor. The catheter’s slideable balloons allow for the exclusion of arterial side branches, precluding therapeutic washout. The increased intra-arterial pressure that occurs upon infusion of gemcitabine into the isolated arterial segment drives the drug across the arterial wall, resulting in targeted, localized delivery to the region surrounding the tumor. Gemcitabine and its metabolites are drained from the pancreas into the splenic vein, then to the liver via the portal vein

Gemcitabine (2’,2’-diflouro-2’-deoxycytidine, dFdC) has been a cornerstone in the treatment of pancreatic cancer for the last three decades. Upon cell entry, the gemcitabine prodrug is phosphorylated into mono-, di-, and triphosphate forms (Fig. 1A; reviewed in [5]). The diphosphate form (dFdCDP) is a potent inhibitor of ribonucleotide reductase that acts to deplete deoxyribonucleotide pools required for DNA synthesis, while the triphosphate form (dFdCTP) blocks DNA synthesis by acting as a chain terminator. The primary inactivation pathway involves cytidine deaminase (CDA), which converts gemcitabine to its inactive metabolite 2’, 2’-difluorodeoxyuridine (dFdU); CDA is expressed at high levels in plasma and the liver (reviewed in [6]) and is overexpressed relative to normal adjacent parenchyma in human pancreatic ductal adenocarcinoma tumors [7].

Due to the desmoplastic and avascular nature of pancreatic tumors, drug penetration is a key limiting factor in therapeutic response, independent of the agent used. Intra-arterial (IA) drug delivery, which aims to selectively increase the drug concentration at the tumor site while reducing systemic exposure, has been used in the treatment of many tumor types, including experimental study with gemcitabine in patients with pancreatic cancer. The effectiveness of IA chemotherapy delivery is influenced by pharmacokinetic factors, including total body clearance and regional exchange rates. Although its short half-life makes gemcitabine a good candidate for IA delivery with reduced systemic toxicity, the abovementioned barriers to tumor penetration remain. In addition to the desmoplastic nature of pancreatic cancer, there is no dedicated blood vessel supplying the pancreas and angiography often reveals a lack of tumor feeder vessels appropriate for drug delivery engagement. In past experimental approaches, a ‘blood unification’ strategy has been utilized for effective IA drug delivery to the pancreas [8, 9]. However, approaches using this strategy are technically demanding and can be associated with end organ ischemic risk due to permanent blood vessel occlusion [8, 9]. These factors limit the benefit of non-selective IA delivery to vessels surrounding the pancreas and its tumors.

Trans-arterial micro perfusion (TAMP) is a novel technique that employs a unique double-balloon endovascular catheter with an adjustable occlusion distance (RenovoCath^®^, RenovoRx, Mountain View, CA) for targeted drug delivery in the absence of tumor feeder vessels (Fig. 1B). RenovoCath’s slidable balloons allow for catheter positioning with arterial side branch exclusion, thereby precluding therapeutic washout while driving the infusate across the vessel wall via increased arterial pressure [10]. TAMP overcomes the challenges mentioned above, providing effective localized drug delivery while greatly reducing the risk of ischemia by utilizing transient, rather than permanent, vessel occlusion.

Prior clinical studies in patients with LAPC have shown TAMP to be safe with promising clinical efficacy, especially in patients with prior chemoradiation [11, 12]. The ongoing phase 3 TIGeR-PaC study (NCT03257033) is testing the impact of TAMP-mediated localized IA gemcitabine (IAG) delivery versus systemic intravenous gemcitabine (IVG) delivery on overall survival and progression-free survival in patients with LAPC. The analysis presented herein was undertaken as a TIGeR-PaC sub-study to establish the pharmacokinetics (PK) of gemcitabine and its inactive metabolite, dFdU, with TAMP-mediated localized IAG versus systemic IVG. We also examined the relationship between systemic gemcitabine and dFdU levels and the pre-treatment to post-IAG change in CA 19 − 9 tumor marker levels, which serve as a surrogate for tumor response in patients with LAPC.

## Materials and methods

### Ethical considerations

The study was conducted in accordance with the Declaration of Helsinki, Good Clinical Practice, and applicable regulatory requirements. The study protocol was approved by the Institutional Review Board/Independent Ethics Committee at each study site, and written informed consent was obtained from all participants.

### Participants

All patients in this pharmacokinetics and pharmacodynamics sub-study were participants in the TIGeR-PaC phase 3 clinical trial (NCT03257033; protocol available on ClinicalTrials.gov). Main enrollment criteria for the TIGeR-PaC study included age ≥ 18 years with histology- or cytopathology-confirmed diagnosis of LAPC and an Eastern Cooperative Oncology Group (ECOG) Performance Status of 0 or 1. Sub-study participants provided consent for data collection, analysis, and publication, and were selected from TIGeR-PaC study sites that agreed to participate. Planned sub-study enrollment included a minimum of 10 IAG patients (5 treated via the celiac axis and 5 via the SMA) and 5 IVG patients (after attrition).

### Treatment

Participants were randomized to IAG or IVG treatment as part of the TIGeR-PaC study. Participants in the IAG group received localized IA gemcitabine infusions via TAMP at 1000 mg/m^2^ over 20 min at an infusion rate of 6 mL/minute; total time for which the catheter was in place with balloons inflated was approximately 25 min. Participants in the IVG group received systemic intravenous (IV) gemcitabine infusions at 1000 mg/m^2^ over 30 min.

### Sample collection

Blood samples for pharmacokinetic analyses were collected from a peripheral vein proximal to the infusion venous circuit in all patients. For the IAG group, blood samples were collected 5 min before the onset of localized IA infusion, and at 10, 15, 20, 30, 40, 60, and 90 min after the onset of infusion. For the IVG group, blood samples were collected 5 min before the onset of systemic IV infusion, and at 10, 20, 30, 40, 50, 70, and 100 min after the onset of infusion. Venous blood was collected in vacutainer tubes containing a tetrahydrouridine solution. Plasma from each sample was frozen and shipped to the reference lab (Johns Hopkins Analytical Pharmacology Core Laboratory, Baltimore, MD) for analysis.

### Gemcitabine and dFdU assays

Frozen plasma samples were prepared and analyzed by liquid chromatography (LC)/mass spectrometry (MS) according to the reference lab’s standard operating procedures for the quantitation of gemcitabine and 2’, 2’-difluoro-2’-deoxyuridine. Each analytical run included a standard calibration curve, matrix blank, matrix blank + internal standard, and 3 levels of quality control samples. Sample extracts were subjected to reverse phase ultra-high performance liquid chromatography (UPLC; Shimadzu Corporation, Columbia, MD) to separate the analyte from potentially interfering material. The column effluent was analyzed by MS (AB Sciex, Framingham, MA) with introduction via electrospray ionization. Data acquisition was performed using Analyst software (AB Sciex, Marlborough, MA).

### Tumor marker assay

The biological half-life of tumor marker CA19-9 is 1 to 3 days [13]. Thus, CA19-9 levels were assessed before each IAG treatment and 2 weeks after each treatment. CA 19 − 9 assay measurements were performed locally at each participating site’s laboratory.

### Pharmacokinetic analysis

A non-compartmental analysis (NCA) was performed by Momentum Metrix (Dublin, CA) on the collected gemcitabine and dFdU concentration-time data. The analysis was conducted in R (version 4.4.3) using the pkr package.

The maximum concentration achieved (C_max_) following a single dose of gemcitabine was determined by identifying the maximum observed gemcitabine and dFdU concentrations. The area under the concentration curve from time 0 to the last observable concentration (AUC_0−t_) was calculated using the linear-up/log-down methodology. Actual time was used to calculate the AUC_0−t_ for individual concentration-time profiles; samples that were collected prior to onset of infusion were imputed with an actual sampling time of 0.00 h.

The targeted extraction ratio (E_target_) was derived from the absolute bioavailability (F_abs_, the fraction of an administered drug that reaches the systemic circulation from a non-IV route compared to IV) to quantify the fraction of drug extracted at the site of IA delivery, and was calculated as E_target_ = 1 – F_abs_ [14].

### Statistical analysis

Summary statistics (mean, percentage, minimum, maximum) were used to evaluate participant demographics and clinical characteristics. Concentration-time and PK metrics obtained from the NCA were utilized for comparisons of gemcitabine and dFdU exposure by individual patient, group (IAG vs. IVG), and analyte; exposure was summarized using descriptive statistics (mean, median, minimum, maximum, 25th and 75th percentiles). Linear correlations between the AUC_0−t_ of gemcitabine and dFdU and the pre- to post-treatment change in CA 19 − 9 levels were calculated using Pearson’s correlation coefficient; significance testing utilized a two-sided α of 0.05. Participants with normal CA 19 − 9 levels at baseline (*n* = 3) were excluded from the analysis; CA 19 − 9 data were analyzed as the percent change from levels assayed pretreatment to levels assayed at 2 weeks post-IAG procedure.

## Results

### Patients

A total of 16 participants across 6 sites of the TIGeR-PaC Phase 3 clinical trial were enrolled in this PK sub-study, including 11 patients from the IA gemcitabine arm and 5 control patients from the IV gemcitabine arm. All enrolled participants completed the study; i.e., there was no attrition. Mean age was 63.1 years (range, 43 − 84 years) for the IAG group and 67.8 years (range, 65 − 70 years) for the IVG group (Table 1). Of the 16 total participants, 7 were female and 9 were male. Pancreatic tumor size (largest diameter) ranged from 3.1 to 7.0 cm in the IAG group and 1.9–6.1 cm in the IVG group. All participants in the IAG group had received prior SBRT; among the IVG group, 4 patients had received prior SBRT, and 1 patient had received prior intensity-modulated radiation therapy (IMRT).

**Table 1:** Patient Demographics and Pre-Treatment Clinical Characteristics

	IAG Group (*n* = 11)	IVG Group (*n* = 5)	Total (*N* = 16)
Sex, *n* (%)			
Female	6 (54.5%)	1 (20.0%)	7 (43.8%)
Male	5 (45.5%)	4 (80.0%)	9 (56.2%)
Age in years, mean (min, max)	63.1 (43, 84)	67.8 (65, 70)	64.6 (43, 84)
Tumor location, n (%)			
Pancreas head	4 (36.4%)	1 (20.0%)	5 (31.2%)
Pancreas body	7 (63.6%)	4 (80.0%)	11 (68.8%)
Tumor size in cm, median (min, max)	3.6 (3.1, 7.0) [1]	4.1 (1.9, 6.1)	3.6 (1.9, 7.0)
Treatment artery, n (%)			
Celiac trunk	5 (45.5%)	N/A	−
Superior mesenteric artery	6 (54.5%)	N/A	−
Prior radiation, n (%)			
SBRT	11 (100%)	4 (80.0%)	15 (93.8%)
IMRT	0 (0.0%)	1 (20.0%)	1 (6.2%)

IAG = intra-arterial gemcitabine; IMRT = intensity modulated radiation therapy; IVG = intravenous gemcitabine; max = maximum; min = minimum; N/A = not applicable; SBRT = stereotactic body radiation therapy; SD = standard deviation [1] Tumor size data available for 10 of the 11 patients in the IAG group

### Pharmacokinetics

**Fig. 2:**
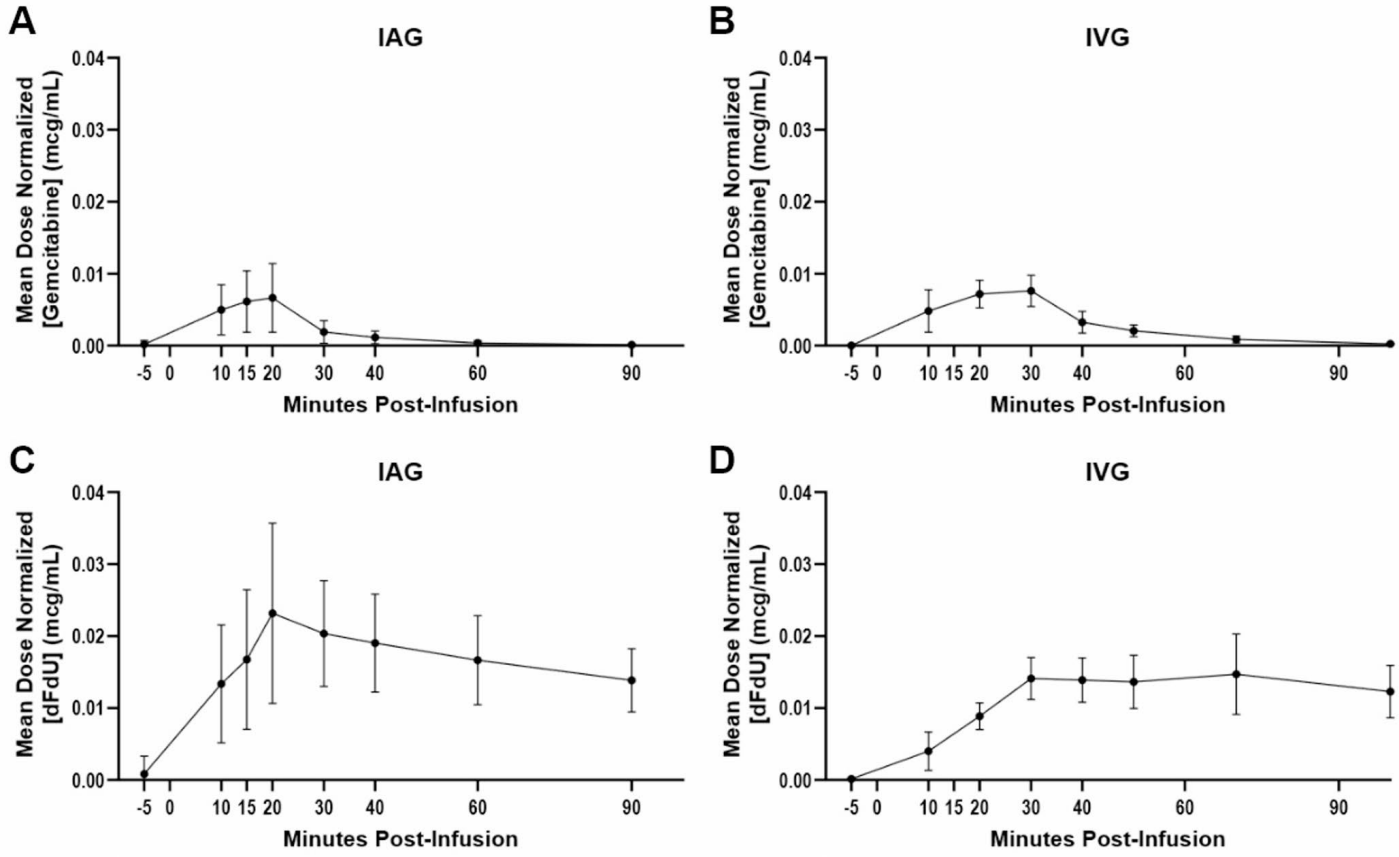
Mean dose normalized concentrations over time of gemcitabine ([**A**] IAG, [**B**] IVG) and dFdU ([**C**] IAG, [**D**] IVG); error bars represent one standard deviation

**Fig. 3:**
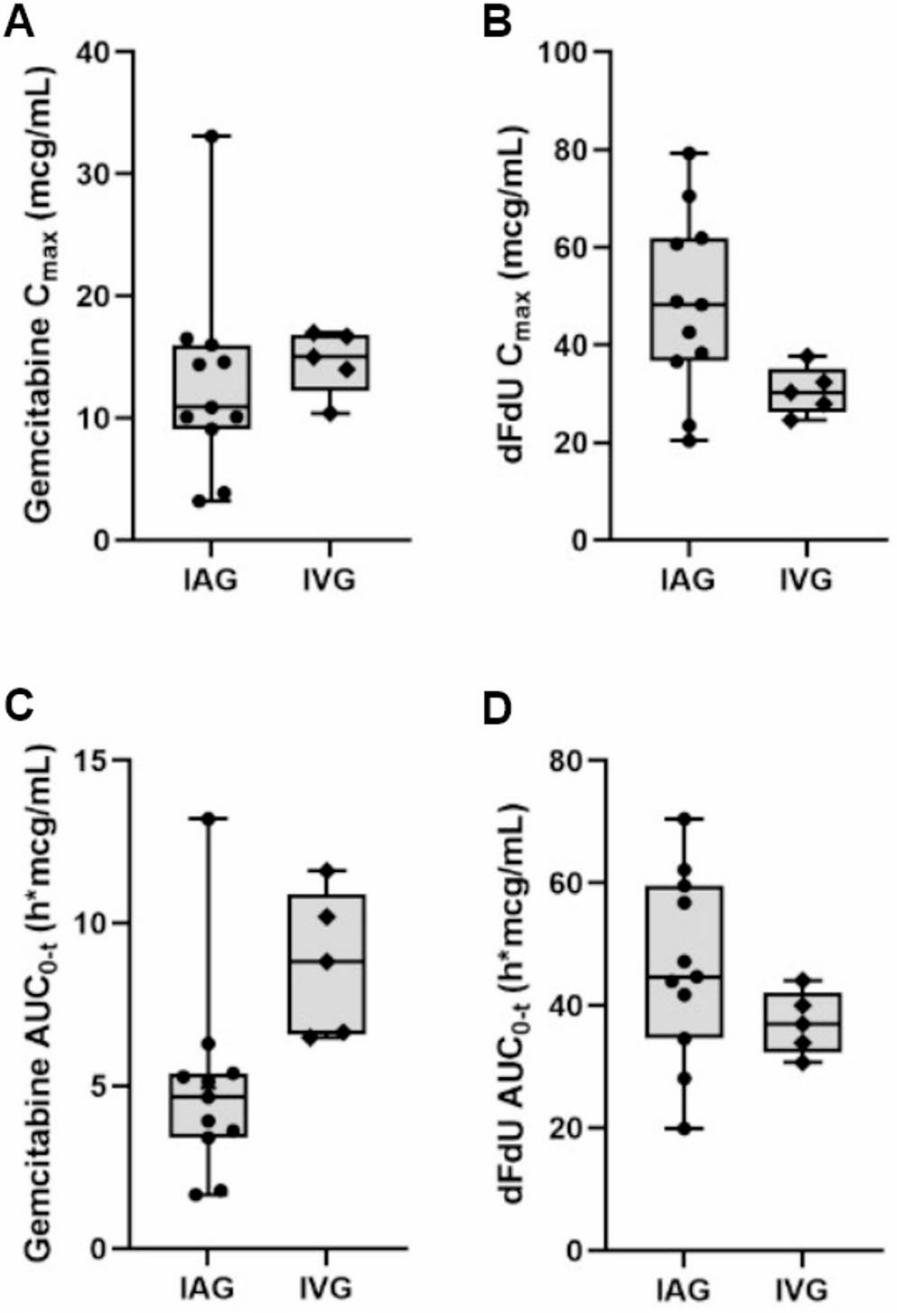
Box-and-whiskers plots of gemcitabine and dFdU pharmacokinetic parameters showing minimum and maximum (error bars), median (horizontal line), and 25th and 75th percentiles (bottom and top of bars, respectively). (**A**) The C_max_ for gemcitabine is lower with IA (median: 10.9 mcg/mL; min, max: 3.2, 33.1 mcg/mL) vs. IV (median: 15.0 mcg/mL; min, max: 10.4, 17.0 mcg/mL) administration, despite a 50% higher drug concentration during infusion (50 mg/minute/m^2^ vs. 33.3 mg/minute/m^2^, respectively). (**B**) Conversely, the C_max_ for dFdU was higher with IA (median: 48.3 mcg/mL; min, max: 20.4, 79.3 mcg/mL) vs. IV (median: 30.3 mcg/mL; min, max: 24.6, 37.7 mcg/mL) administration, consistent with rapid conversion of gemcitabine to its inactive metabolite. (**C**) Total systemic gemcitabine exposure (AUC_0−t_) was lower with IA (median: 4.7 h*mcg/mL; min, max: 1.7, 13.2 h*mcg/mL) compared to IV (median: 8.8 h*mcg/mL; min, max: 6.5, 11.6 h*mcg/mL) administration. (**D**) In contrast, the AUC_0−t_ for dFdU was higher for IA (median: 44.7 h*mcg/mL; min, max: 19.9, 70.5 h*mcg/mL) versus IV (median: 37.0 h*mcg/mL; min, max: 30.7, 44.1 h*mcg/mL) administration

Gemcitabine and dFdU C_max_ and AUC_0−t_ for localized IA versus systemic IV administration of gemcitabine are shown in Table 2. Gemcitabine was rapidly metabolized following both IA and systemic IV administration (Fig. 2). Blood levels of dFdU, which is excreted through the kidneys, were higher and more persistent compared to gemcitabine with both administration routes. The C_max_ for gemcitabine was lower with localized IA delivery versus systemic IV administration despite a 50% higher concentration during IA infusion (50 mg/minute/m^2^ versus 33.3 mg/minute/m^2^) (Fig. 3A). Conversely, the C_max_ for dFdU was higher with IA versus IV administration (Fig. 3B), consistent with rapid conversion of gemcitabine to its inactive metabolite. Total gemcitabine systemic exposure (AUC_0−t_ ) was lower with localized IA delivery versus systemic IV administration (Fig. 3C). In contrast, systemic exposure to its inactive metabolite, dFdU, was higher with IA versus IV gemcitabine administration (Fig. 3D). The absolute bioavailability (Fabs) of gemcitabine delivered IA vs. IV was 0.489, resulting in a targeted extraction ratio (E_target_) of 0.511 (Table 2).

**Table 2:** Targeted Extraction Ratio of Gemcitabine Delivered Intra-arterially vs. Intravenously

	IAG Group AUC_0−t_/Dose (*N* = 11)		IVG Group AUC_0−t_/Dose (*N* = 5)			
	GM	CV%	GM	CV%	F (IAG/IVG)	E_target_ (1 – F)
Gemcitabine	2.13	70.9	4.36	34.3	0.489	0.511

CV% = geometric coefficient of variation; E_target_ = targeted extraction ratio; F = absolute bioavailability; GM = geometric mean; IAG = intra-arterial gemcitabine; IVG = intravenous gemcitabine

We also examined whether the treatment site utilized for localized IA gemcitabine delivery (i.e., SMA versus celiac axis) affected PK parameters. No differences were observed in C_max_ or AUC, for either gemcitabine or dFdU, following delivery via the SMA versus the celiac artery (Table 3).

**Table 3:** Gemcitabine and Difluorodeoxyuridine Pharmacokinetic Parameters

	Gemcitabine				dFdU		
	IAG Group (*n* = 11)	IVG Group (*n* = 5)	*P*-value	IAG Group (*n* = 11)		IVG Group (*n* = 5)	*P*-value
C_max_, mean (SE) (mcg/mL)	12.9 (2.42)	14.6 (1.19)	0.535	48.3 (5.61)		30.6 (2.20)	0.012
AUC_0−t_, mean (SE) (h*mcg/mL)	4.9 (0.94)	8.8 (0.99)	0.018	46.3 (4.59)		37.1 (2.33)	0.097
**IAG Group (n = 11)**:	**SMA** **(n = 6)**	**Celiac** **(n = 5)**	***P*** **-value**	**SMA** **(n = 6)**		**Celiac** **(n = 5)**	***P*** **-value**
C_max_, mean (SE) (mcg/mL)	8.3 (2.74)	16.6 (4.22)	0.141	47.4 (9.79)		49.3 (5.34)	0.873
AUC_0−t_, mean (SE) (h*mcg/mL)	3.7 (0.75)	6.4 (1.72)	0.201	45.0 (7.91)		47.9 (4.57)	0.752

AUC_0−t_ = area under the drug plasma concentration curve from time zero to the last measured concentration; C_max_ = maximum plasma drug concentration; dFdU = difluorodeoxyuridine; h = hours; SE = standard error of the mean; SMA = superior mesenteric artery

### Pharmacodynamics

**Fig. 4:**
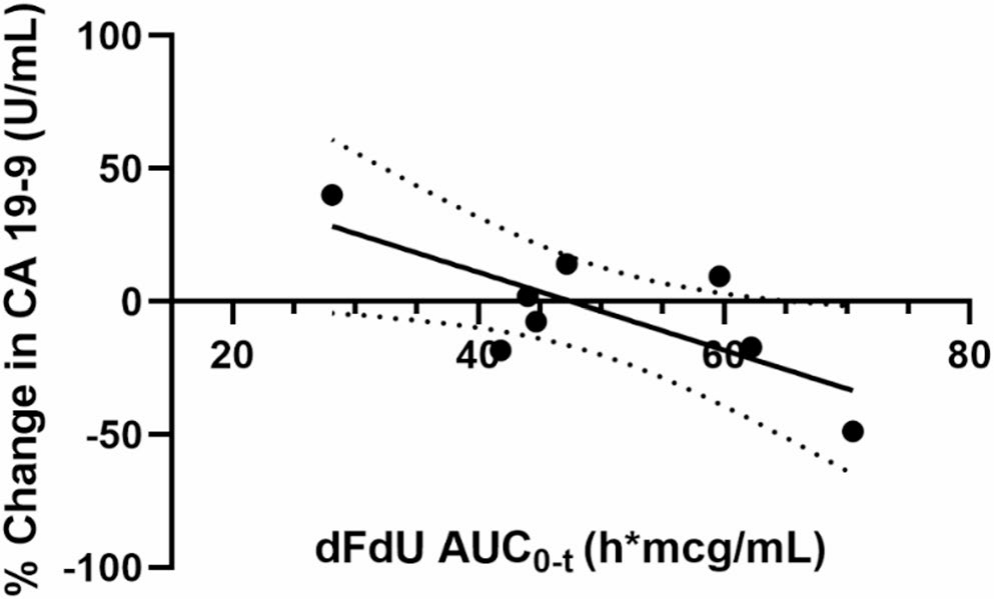
Correlation between systemic dFdU levels and change in CA 19 − 9 levels. With IAG, there was a significant correlation between dFdU AUC_0−t_ and the percent decrease from pre-treatment to post-IAG in CA 19 − 9 tumor marker levels (Pearson’s *r* = 0.75; *P* = 0.034)

We examined the relationship between total gemcitabine or dFdU exposure and the pre- to post-treatment change in CA 19 − 9 tumor marker levels with IA gemcitabine delivery. There was a modest correlation between a reduction in CA 19 − 9 tumor marker levels and systemic gemcitabine exposure (Pearson’s *r* = -0.39; *P* = 0.334). A stronger correlation was observed between a reduction in CA 19–19 levels and dFdU exposure (Pearson’s *r* = -0.75; *P* = 0.034) (Fig. 4), with higher dFdU levels corresponding to larger reductions in CA 19 − 9 levels following TAMP-mediated IAG treatment. The differences between gemcitabine and dFdU in this correlation with CA 19 − 9 levels could be due to (1) the clear outlier in the IAG dataset, and (2) the longer half-life of dFdU which attenuates the variability of single measurements. In addition, gemcitabine levels may represent incomplete systemic escape through local microvasculature despite localized delivery.

## Discussion

Regional drug delivery aims to enhance chemotherapy selectivity by increasing the drug concentration at the tumor site while reducing systemic exposure and unwanted off-target effects by drug removal via first pass through the target region prior to systemic circulation.

IA drug delivery is one of several regional methods (intra-peritoneal [IP], and intrathecal [IT] delivery being others) where the overall selectivity for regional administration is defined by the ratio of target site concentration to systemic concentration. The effectiveness of regional delivery is influenced by pharmacokinetic factors, including total body clearance and regional exchange rates. Specifically, the faster the drug is eliminated by the rest of the body and the smaller the blood perfusion rate to the tumor, the greater the advantage of IA drug delivery.

With its short half life and high clearance rate, gemcitabine remains a favorable drug for localized delivery. As such, it has been proposed and used successfully to treat solid tumors, including regional delivery for bladder cancer and lung cancer. However, targeting regional delivery to pancreatic cancer has been a challenge due to the lack of dedicated blood vessels to the organ and the avascular nature of pancreatic tumors. In the past, nonselective arterial infusion of gemcitabine to patients with pancreatic cancer has provided modest clinical improvement [15]. Furthermore, techniques to “unify” the blood supply to pancreatic tumors pose technical challenges and have resulted in limited success [8, 9, 16]. A key limitation in these approaches remains large perfusion to non-target organs such as the liver and/or spleen rather than the pancreas, forgoing the advantages of localized delivery. The technique presented herein, in which a double-balloon occlusion catheter is used to achieve TAMP, has the potential to overcome these limitations by (1) forgoing non-selective perfusion to distal organs (i.e., liver), and (2) achieving a low perfusion rate to target tissue (6 mL/minute in the present study).

In the present study, TAMP-mediated IA gemcitabine delivery in TIGeR-PaC study patients with LAPC demonstrated pharmacokinetics consistent with a substantial first-pass extraction at the site of IA delivery. Following TAMP administration, gemcitabine exhibited rapid plasma clearance similar to the IV route. However, both peak plasma gemcitabine concentration (C_max_) and total systemic exposure (A_0−t_) were markedly lower with TAMP-mediated IAG, indicating that a significant fraction of the IA-delivered dose was retained locally rather than reaching the systemic circulation. As F_abs_ (absolute bioavailability) represents the fraction of IA-delivered drug that escapes into the systemic circulation, a lower F_abs_ reflects greater local drug retention at the target site – the desired pharmacologic outcome of TAMP delivery. The observed E_target_ of 0.511 indicates that approximately 51% of the IA-delivered gemcitabine was extracted at the tumor site before reaching systemic circulation. This was shown to be the case previously in a preclinical porcine model in which a 100-fold increase in local gemcitabine tissue concentration was achieved with TAMP-mediated delivery versus IV administration [10]. The decreased systemic exposure of the active compound observed in the IAG group should translate to a favorable reduction in systemic side effects when the standard dose of gemcitabine (1000 mg/m^2^) is delivered via a localized, IA route versus a systemic IV route.

Of note, dFdU levels were increased with TAMP-mediated IAG versus systemic IVG, potentially suggestive of gemcitabine being metabolized locally prior to reaching systemic circulation. Indeed, it is well established that CDA, responsible for converting gemcitabine to dFdU, is overexpressed in pancreatic tumors and contributes to gemcitabine resistance [7, 17, 18]. Our results contrast with prior studies in which dFdU levels following unselective hepatic arterial gemcitabine infusion were either lower or unchanged from those with systemic infusion [19]. These observations are supportive of a distinct drug-tissue exchange rate along with localized gemcitabine metabolism following TAMP, as this route of delivery targets tissue near the site of arterial delivery rather than the liver.

The C_max_ for gemcitabine and dFdU among the IVG group was consistent with that of previously published studies. In the current study, the mean peak plasma concentrations among the IVG group were 14.6 mcg/mL for gemcitabine and 30.6 mcg/mL for dFdU. In comparison, in studies in which 1000–1250 mg/m^2^ gemcitabine was administered intravenously over 30 min, peak plasma concentrations ranged from 10 to 40 mcg/mL for gemcitabine and 28 to 52 mcg/mL for dFdU [20].

In this study, there was a significant correlation between increased dFdU AUC_0−t_ levels and a pre-treatment to post-IAG reduction in CA 19 − 9 tumor marker levels, a biomarker for therapeutic response in our patient population. Among participants in the IAG group, we observed a CA 19 − 9 reduction of up to 40% after a single treatment; dFdU, with its long half life and excretion by the kidneys rather than the liver, may be a better harbinger of localized drug delivery into the tumor tissue than serum gemcitabine levels and, thus, better correlated with therapeutic response as measured by CA 19 − 9 in our studies. Reductions in CA 19 − 9 levels have been shown to predict improved survival following tumor resection in patients with pancreatic cancer [21, 22]. The observed correlation between dFdU AUC_0-t_ levels and pre-treatment to post-IAG reductions in CA 19 − 9 levels may be indicative of improved tissue penetration with IAG. Results of the ongoing phase 3 TIGeR-PaC study will help determine whether this translates into improved survival and reduced toxicity with IAG versus IVG. Of note, a significant correlation was not observed between serum gemcitabine levels and a reduction in CA 19 − 9 levels in our patient cohort. Possible reasons for this lack of correlation include (1) systemic gemcitabine levels with IA delivery may actually represent systemic escape of the drug into the circulation via the microvasculature despite localized delivery, and (2) technical factors may have occurred during the study, as indicated by the obvious outlier among our relatively small cohort with high gemcitabine C_max_ and AUC_0−t_ levels (see Figs. 2 and 3).

## Conclusions

In summary, localized IAG delivery resulted in decreased systemic gemcitabine levels and increased systemic dFdU levels compared to IVG delivery. A significant correlation between increased dFdU levels and a decrease from baseline in CA 19 − 9 levels – a surrogate for tumor response – was observed with IAG. Thus, in addition to providing increased local potency, the IAG approach, in which gemcitabine is rapidly converted to its inactive metabolite dFdU, may also be beneficial in decreasing gemcitabine-related systemic side effects while providing improved outcomes in patients with LAPC.

## Data Availability

The data generated in this study are available for non-commercial use upon request from the corresponding author.
